# Knee kinesiography in 2026: Current and future applications for digital orthopaedics and personalised total knee arthroplasty: A narrative review

**DOI:** 10.1002/jeo2.70674

**Published:** 2026-02-27

**Authors:** Clément Favroul, Alix Cagnin, Cécile Batailler, Sébastien Lustig

**Affiliations:** ^1^ Department of Orthopaedics Surgery and Sports Medicine, FIFA Medical Center of Excellence, Croix‐Rousse Hospital, Hospices Civils de Lyon Lyon North University Hospital Lyon France; ^2^ Emovi Montreal Quebec Canada; ^3^ Univ Lyon, Claude Bernard Lyon 1 University, IFSTTAR, LBMC UMR_T9406 Lyon France

**Keywords:** biomechanics, KneeKG, rehabilitation, robotic‑assisted surgery, total knee arthroplasty

## Abstract

Knee Kinesiography, using the KneeKG system, delivers clinically feasible, weight‐bearing (WB) three‐dimensional knee kinematics and is shifting total knee arthroplasty (TKA) from static templating to function‐focused personalisation. This narrative review synthesises how KneeKG applications changed pre‐ and postoperative care and how these changes can translate into better patient outcomes. Preoperatively, KneeKG quantifies dynamic alignment, varus thrust, dynamic flexion contracture, pivot pattern, tibiofemoral translation, and tibial rotation during gait, defining a functional profile that relates to symptoms and can inform alignment philosophy, bearing selection, and soft‐tissue strategy. When coupled with navigation or robotics, these data allow the anticipation of mid‐flexion balance requirements while enabling surgeons to set functional targets more likely to yield natural, stable knee in WB condition. A KneeKG‐guided prehabilitation pathway also facilitates the recalibration of expectations before surgery and can improve early patient‐reported outcomes. Postoperatively, KneeKG audits whether intraoperative parameters (e.g., achieved gaps, implant components, etc.) translate into the intended dynamic behaviour, verifies bearing‐intended pivot during WB tasks, and reveals modifiable residual deficits—such as persistent thrust, stiff‐knee gait, valgus collapse, or excessive external tibial rotation—to target with therapy. Function‐focused rehabilitation programmes anchored on a KneeKG exam demonstrated acceleration of recovery, increase in outcome scores, satisfaction, and raise of the proportion of patients reaching a patient acceptable symptom state, with benefits maintained beyond one year in subsets. Across the episode of care, KneeKG provides a common functional language that links preoperative planning, intraoperative execution, and postoperative rehabilitation, allowing teams to iterate from plan to performance and to close the loop between digital precision and real‐world function. By operationalizing dynamic information at the point of care, this exam supports surgeons towards more accurate decisions, more predictable execution, and more personalised rehabilitation; ultimately improving patient‐centred results after TKA.

**Level of Evidence:**

Level V, narrative review.

## Graphical abstract

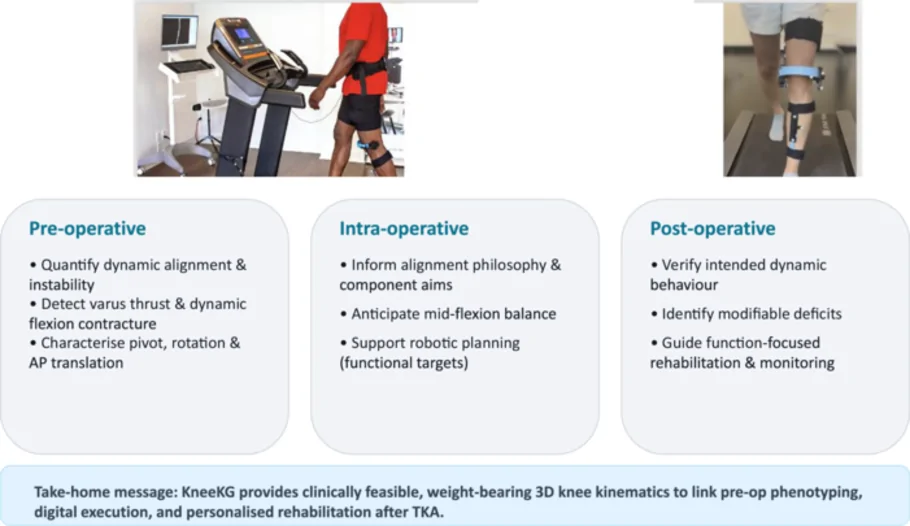

**Abbreviations**
3D: three‐dimensional
AAOS: American Academy of Orthopaedic Surgeons
AP: anteroposterior
CE: Conformité Européenne
CPAK: coronal plane alignment of the knee
CPG: clinical practice guidelines
CS: cruciate‐substituting
FDA: US Food and Drug Administration
F‐F: function focused
FKP: functional knee phenotype
HKA: hip–knee–ankle
JLO: joint line obliquity
KOOS‐JR: Knee injury and Osteoarthritis Outcome Score for Joint Replacement
MP: medial pivot
OA: osteoarthritis
OR: operating room
PASS: Patient Acceptable Symptom State
PCL: posterior cruciate ligament
PROMs: Patient‐Reported Outcome Measures
PS: posterior stabilised
TEA: transepicondylar axis
TKA: total knee arthroplasty
TS: total stabilised
WB: weight bearing

## INTRODUCTION

Despite advances in implant design, surgical technique, and digital surgery, approximately 20% of patients remain dissatisfied after total knee arthroplasty (TKA) [4, 28]. With annual US procedure volumes nearing one million and projected to rise markedly [61], addressing this persistent gap is a priority. Digital surgery has increased precision and standardisation, enabled individualised alignment and balancing strategies, but the persisting challenge remains to define patient‐specific functional targets that translate technical precision into stable motion, especially during weight‐bearing (WB) tasks [65].

Current planning tools are largely static. Long‐leg radiographs provide hip–knee–ankle (HKA) angle and joint‐line obliquity (JLO) for templating, and patient‐reported outcome measures (PROMs) capture the patient's perspective. Neither, however, tells the surgeon how the knee actually behaves under load during activities of daily living. Multiple studies show that static alignment does not reliably predict dynamic behaviour; in a notable proportion of knees, the WB alignment during walking even opposes the standing alignment [13, 30]. As surgeons navigate choices of implant design, alignment philosophy and soft‐tissue strategy across the arc of motion, routine, WB functional data are needed to prioritise among competing parameters for each patient.

The Knee Kinesiography (the generic name of the exam) performed with the KneeKG™ system (Emovi, Montreal, Quebec, Canada) addresses this gap by delivering clinically feasible, WB 3D kinematics measurement during gait and functional tasks. The exam quantifies dynamic alignment and instability through objective markers (i.e., varus thrust, dynamic flexion contracture, pivot pattern, tibiofemoral translation and tibial rotation) which are mechanistically linked to symptoms and function.

Preoperatively, these markers help phenotype TKA candidates, inform alignment and bearing choices, and anticipate balance requirements, including the often‐neglected mid‐flexion range where instability is frequently observed. Used alongside navigation or robotics, KneeKG provides a functional language that interfaces naturally with intraoperative data to set objective targets.

Postoperatively, the same measurement allows teams to audit whether achieved gaps and component parameters translate into the intended dynamic behaviour in vivo (e.g., varus–valgus during stance, pivot during WB tasks, etc.), and reveals modifiable deficits such as persistent thrust, stiff‐knee gait, valgus collapse or excessive external tibial rotation that can be addressed with targeted rehabilitation. Early programmes that anchor prehabilitation or rehabilitation on a KneeKG exam have reported improvements in outcome scores, satisfaction, and higher rates of patient with acceptable symptom state.

Thirteen years after the initial technical review of the system [48], the evidence base now supports a function‐focused pathway in which KneeKG links prehabilitation, planning, intraoperative execution and postoperative care. This narrative review summarises current evidence, highlights pragmatic integration before and after TKA, and outlines how KneeKG can help convert digital precision into real‐world function and patient‐centred outcomes.

## THE KNEEKG SYSTEM

### Design and validation

**Figure 1:**
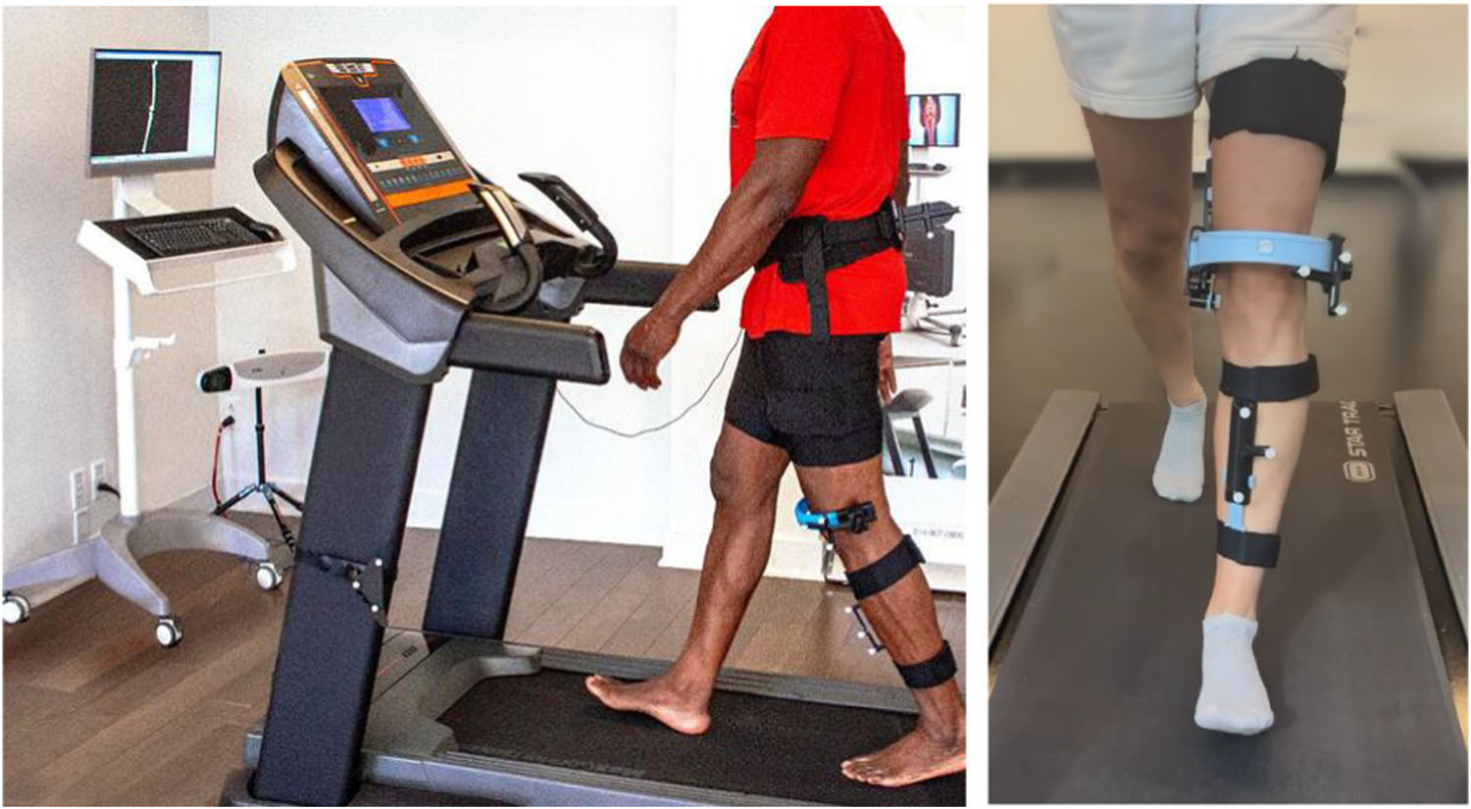
A knee kinesiography exam conducted in clinic where the KneeKG system is secured on a patient's lower limb.

**Figure 2:**
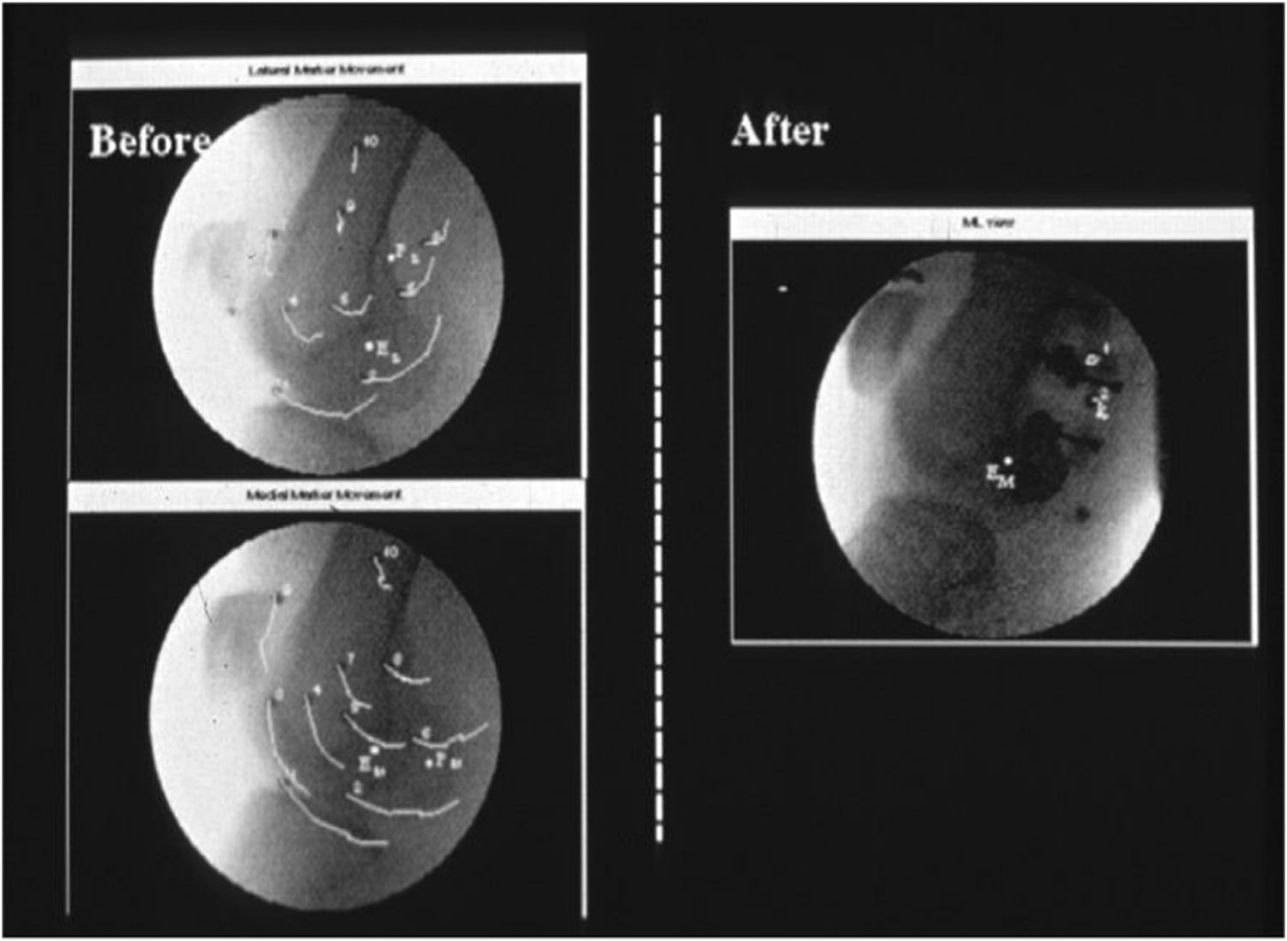
The reduction of soft‐tissue artefacts with respect to the underlying bone between skin‐mounted markers (“Before”) and the exoskeleton of the KneeKG system (“After”) during an active knee flexion on fluoroscopic images.^27^

The Knee Kinesiography exam is performed with the KneeKG system, a class II medical device (FDA 510 K cleared and Health Canada licensed) and relies on optical tracking (NDI, Northern Digital Inc., Canada) to record 3D knee kinematics during functional tasks such as treadmill walking, squats or lunges, as well as non‐WB active or passive flexion. The KneeKG is the only commercially available device to perform this exam in clinic by a trained technician installing the adjustable exoskeleton equipped with reflective markers secured to the thigh and shank (Figure 1). By constraining relative motion between markers and bony segments, the exoskeleton markedly limits soft‐tissue artefacts; a fluoroscopic study reported an error reduction of about six‐fold versus skin‐mounted markers [27] (Figure 2).

A standard workflow takes overall 15–20 min: patient familiarisation at comfortable gait speed, device positioning, anatomical landmarking, registration, and data capture (typically two 45‐s gait trials plus additional tasks according to protocol). Registration mirrors navigation/robotic workflows: the hip centre is estimated by circumduction; the ankle centre as the midpoint of the malleoli; and the knee centre as the midpoint between the epicondyles, projected onto the flexion–extension axis derived from active flexion–extension. Epicondyles and malleoli are digitised with a pointer non‐invasively. The system provides accurate, repeatable measures of flexion–extension, varus–valgus and internal–external tibial rotation, and quantifies tibiofemoral AP translation [32, 41, 62].

### Clinical use

The KneeKG provides continuous 3D kinematic waveforms (i.e., flexion/extension, varus/valgus, and internal/external tibial rotation, tibiofemoral AP translation) and computes derived, clinically relevant markers (e.g., event‐specific angles, amplitudes and peaks) summarising WB functional behaviour, including dynamic HKA alignment, varus thrust, dynamic flexion contracture, and tibial rotation relative to the femur. These markers are clinically meaningful. For example, varus thrust (lateral thrust; a transient increase in varus during loading) is associated with cartilage damage and a two‐ to four‐fold higher risk of medial tibiofemoral osteoarthritis progression [22, 63, 66]. The exam provides dynamic functional information in both non‐WB and WB conditions rather than static alignment alone, unlike CT or radiographs. Despite the limited correlation between static imaging and patients' symptoms, surgeons still rely on radiographs for TKA planning [14]. Static alignment measures are practical but have been shown to be unreliable predictors of dynamic alignment [30]. Consistently, intraoperative navigation during passive non‐WB motion revealed heterogeneous HKA behaviour across the flexion–extension arc, with frontal malalignment that may increase, decrease, or even reverse direction (valgus to varus, or vice versa) [20, 44]. The same phenomenon is observed under WB with KneeKG, where approximately 22% of knees display a dynamic HKA during walking that is opposite to their standing alignment (“switchers”; e.g., static valgus becoming varus dynamically) [13].

These data are directly relevant to knee arthroplasty and contemporary robotic surgery. Preoperative functional HKA alignment measured with KneeKG was demonstrated to be equivalent to radiographic alignment and to intraoperative values recorded by robotic systems [21], a result replicated in a large healthy cohort [42] that supports interoperability across systems. Beyond this concordance, dynamic measures from the exam improve clinical discrimination: they correlate more strongly with patient‐reported outcome measures than radiographs in osteoarthritis, help identify distinct kinematic phenotypes among candidates for TKA, and provide mechanistic insight in cases of unexplained postoperative pain [1, 6, 50, 51].

## IMPROVE TKA PLANNING AND INTERVENTION

### A sensitive assessment of WB knee function

Although the goal of TKA is to restore function, the functional impact of surgery is rarely assessed under load, where patients spend most of their active time and often experience pain and limitations [9]. In recent years, the KneeKG system has been used to characterise in vivo differences between implants and techniques. During gait, AP translation was similar with cruciate‐substituting and posterior‐stabilised designs, supporting the use of cruciate‐substituting inserts to restore kinematics without a femoral box [24]. In revision surgery, semi‐constrained prostheses showed kinematic patterns comparable to primary posterior‐stabilised implants [3]. Preserving or partially titrating the posterior cruciate ligament with an asymmetric ultracongruent bearing minimised deleterious AP translation and internal tibial rotation [49]. Alignment philosophy also matters: kinematic alignment reproduced healthy‐like sagittal, frontal and transverse plane kinematics better than mechanical alignment at 3 years [8].

All these studies indicate that KneeKG–derived functional data are sufficiently sensitive to evaluate surgical interventions (including implant selection and technique) and that these data also correlate with intraoperative measures. Following recommendations from Dasa et al. [19], subsequent proceedings examined associations between KneeKG kinematic metrics and parameters from digital surgical systems. Moderate to very strong correlations were identified between robotic intraoperative parameters and KneeKG kinematics measured after TKA ( | *r* | =0.502–0.815, all 0.001 ≤ *p* ≤ 0.08). Smaller medial laxity and greater lateral laxity at 90° of flexion were associated with significantly higher dynamic varus alignment post‐TKA with MAKO (MAKO 1.0; Stryker, United States) [47]. These results are consistent with a recent fluoroscopic study showing similar relationships, particularly for medial laxity gap in flexion and femoral component alignment with in vivo dynamic varus–valgus during WB tasks after TKA [34].

Analyses with an imageless handheld platform (CORI System, Smith & Nephew, UK) reported similar relationships [53, 54]. Laxity measured in mid‐flexion (30°–60°) showed moderate‐to‐strong correlations with postoperative dynamic frontal‐plane behaviour ( | *r* | =0.495–0.857; all 0.003 ≤ *p* ≤ 0.08). Greater medio‐lateral asymmetry in the achieved medial and lateral gaps at these angles was associated with increased stance‐phase varus–valgus instability and with varus thrust. A shift toward higher medial laxity from extension to flexion was also related to greater postoperative instability during stance (*r* = 0.654; *p* = 0.03), indicating that knees becoming “looser” medially during intraoperative flexion tend to be dynamically less stable after TKA. Together, these observations support assessing balance across the full arc of motion (particularly mid‐flexion) in addition to the traditional checks at full extension and 90° [23].

### Refine influential concepts

Knee pivot motion is an influential parameter for optimising outcomes after TKA. There is broad agreement on a medial pivot in deep flexion (in passive motion and during functional tasks), but reports diverge at lower flexion angles, particularly under WB [25, 50, 52]. This divergence may stem from differences in measurement techniques, variability in patient morphology, or inconsistencies in the definition of pivot patterns. Fluoroscopic studies in healthy knees often show that a lateral pivot was possible in both non‐WB and WB motion, with medial pivot observed dynamically during a limited amount of time (i.e., less than 25% of the stance phase of gait) [34, 39, 40]. Using intraoperative digital data, Seito et al. suggested that preoperative pivot behaviour may inform bearing selection: knees that already display a medial‐pivot pattern may not require a medial‐pivot bearing to achieve medial‐pivot kinematics postoperatively, whereas knees without medial‐pivot motion preoperatively may benefit from such a bearing if that pattern is desired [60].

**Figure 3:**
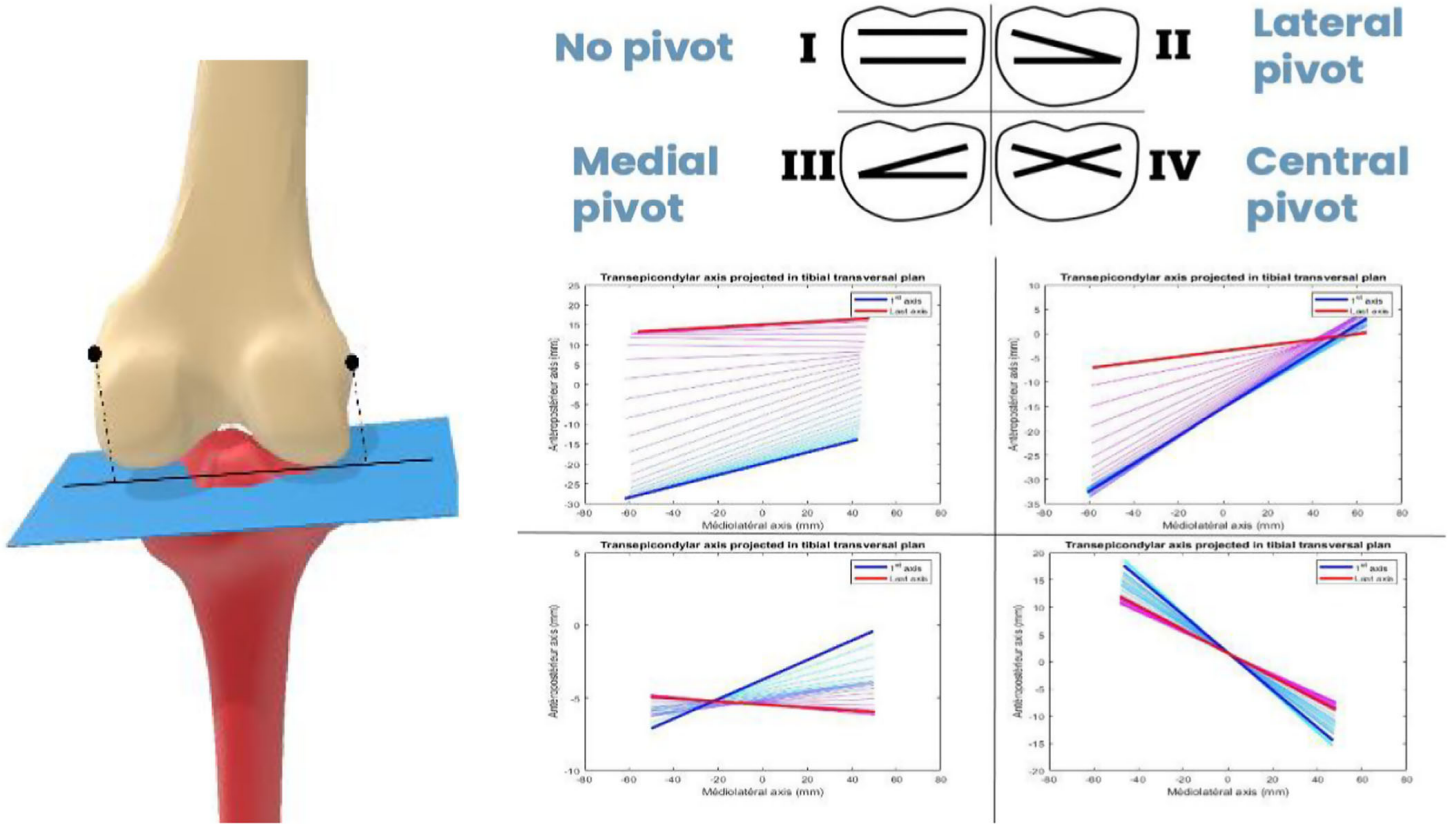
The TEA is projected onto the tibial plateau throughout motion. The knee centre of rotation can be identified by the intersection of consecutive TEA projections and categorised as medial, central, lateral, or no pivot (i.e., pure translation). TEA, transepicondylar axis.

Numerous proceedings investigated the ability of assessing knee pivot motion with the KneeKG, routinely, non‐invasively, and without the cost and radiation from fluoroscopic imaging. Courteille et al. adapted a method proposed by Banks et al. projecting the transepicondylar axis (TEA) tracked with the device onto the tibial plateau plane throughout motion (Figure 3) [2, 18]. In healthy individuals, this approach reported similar results compared with fluoroscopy studies [33]. This method further demonstrated its ability to verify that the motion intended by a specific bearing (i.e., dual‐pivot, Enovis ‐ DJO Global, US) was effectively generally reproduced post‐TKA during WB activity [17]. Finally, the pivot quantified from KneeKG measurement showed promising associations with PROMs post‐TKA, more specifically the importance of exhibiting a multi‐pivot pattern during gait to maximise outcomes [16]. The potential impact of preoperative pivot pattern on post‐TKA outcomes with an ultracongruent bearing also supports the assessment of knee pivot motion before surgery to individualise both implant selection and intervention as it may impact patient satisfaction [16].

Knee phenotyping has gained traction to facilitate decision‐making based on anatomy and laxity. The coronal plane alignment of the knee (CPAK) classification has limitations, with studies reporting weak associations with outcomes after arthroplasty [7]. Hirschmann et al. therefore proposed functional knee phenotypes that include bony morphology and laxities [37]. These functional knee phenotypes (FKPs) based on patient‐specific tibial and femoral orientations showed great potential to classify patients and better understand their characteristics prior to surgery. The addition of functional data, like kinematic markers provided by the KneeKG, allowed to go beyond this static classification and observe kinematic differences between FKPs in healthy subjects [36]. These findings converge with the work of Bensaddek et al. who demonstrated that phenotyping OA patients could be achieved based on functional data from the KneeKG, with the identification of distinct 3D kinematic profiles (which were further linked to patients' clinical condition) [5]. Pursuing these analyses to combine morphological, functional, and kinematic data may help define individualised targets for the most prevalent FKPs to help deliver desired function postoperatively, based on preoperative assessment.

### Integrating preoperative patient‐specific functional data

The future of TKA lies in integrating patient‐specific joint characteristics (geometry, laxity and kinematics) into surgical decision‐making to restore function and improve patient satisfaction. A review from the American Academy of Orthopaedic Surgeons (AAOS) on preoperative planning already advocated incorporating kinematics and functional data that describe dynamic behaviour (e.g., presence of varus thrust or hyperextension), as they inform stability and procedural planning [63].

With the expansion of robotic digital systems, this resonates with recent results exploring the reported associations between preoperative KneeKG measures and intraoperative robotic parameters. Patients exhibiting greater preoperative dynamic varus showed larger lateral laxity in extension and smaller medial laxity in flexion (on average and relative to the lateral side) when assessed intraoperatively before any bone resections [46, 55]. Knees with greater dynamic varus preoperatively were also tighter medially as they flexed intraoperatively (extension laxity > flexion laxity; |*r* | =0.548–0.639; all *p* ≤ 0.01), and preoperative dynamic alignment appeared to significantly influence soft‐tissue behaviour throughout passive motion from 0° to 90° (particularly at 30°–60°; |*r* | =0.705–0.878; all *p* ≤ 0.05). Preoperative varus thrust and dynamic knee flexion contracture may also have an influence on TKA decision and planning process, as they are both associated with excessive soft‐tissue contracture [58]. This better understanding of relationships between technology‐enabled intraoperative measures and objective knee function supports the added value of combining these tools to move towards more optimal surgical planning, improving initial implant positioning and helping anticipate at best what will be observed in the operating room (OR). By reducing unexpected events during surgery, this can help limit the surgeon's movements in the OR and also constitute an advantage for surgeons to use digital surgical systems which have mainly been shown to be beneficial for patients' outcomes.

Integrating preoperative functional data can also improve control over what a surgical plan will deliver. Promising results from a recent study showed that combining preoperative 3D kinematics (such as mean dynamic varus‐valgus alignment during stance) measured by KneeKG and planned intraoperative laxities can significantly enhance the prediction of the dynamic varus‐valgus behaviour after TKA (in statically aligned varus knees) [45]. The difference was significant, as predictive models went from 36% of explained variance (*R* ^2^) without preoperative functional data to 85% when they were included (*p* = 0.003). Surgeons would benefit to be informed that if they aim for an increased medial laxity and/or smaller lateral laxity in flexion, this may deliver more dynamically varus aligned knees during gait in patients with preoperative dynamic varus knees. This approach of function‐focused (F‐F) digital surgery, integrating preoperative kinematic data, may constitute a new era by truly enabling contemporary robotic surgery to personalise TKA interventions to restore function by defining patient‐specific targets for surgeons in the OR.

Finally, a preoperative kinematic assessment with the KneeKG allows the identification of patients at risk of poor outcomes. For example, patients who did not correct their preoperative varus thrust after surgery reported significantly poorer outcomes compared to patients who did not present with a varus thrust on their KneeKG exam post‐TKA [31]. Such patients may be identified before surgery for extra‐focus on the correction of this functional deficiency, with TKA and/or targeted rehabilitation. Another benefit is to generate simple information to educate patients prior to their surgery. Knowing that preoperative expectations are a major contributor to the level of satisfaction, aligning patients' and surgeons' expectations on the functional impact of TKA may increase the likelihood of better outcomes. This is supported by preliminary results on a personalised prehabilitation programme guided by a KneeKG exam 8 weeks before surgery. Patients who benefited from the exam, an education session, and a home‐based exercise programme based on their identified functional deficiencies reported significant improvements at the time of surgery in terms of KOOS‐JR (+15.4%, *p* = 0.02; 59.1 vs. 50.2 in controls) but also a recalibration of their expectations following the tailored exercises and education (30% of expectations were modified: 17% reduced, 13% increased). Expectations regarding improved ability to walk, perform daily activities and climb stairs were the most frequently increased while participating in recreational activities and sports were frequently reduced, highlighting the value of preoperative functional data as an educational aid and supporting tool to better prepare patients for their surgery.

### Enhancing personalised postoperative management

Aligned with value‐based care, postoperative strategies informed by KneeKG have the potential to improve outcomes while using resources efficiently. Multiple studies demonstrate persistent differences between healthy asymptomatic knees and those after TKA, indicating that natural kinematics are not fully restored despite advances in implant design and robotics [10, 43]. In addition to the detrimental presence of varus thrust post‐TKA, surgery may resolve static flexion contracture, but it does not warrant restoration of residual flexion contracture during WB activities, with studies showing its presence 12 months after surgery [51]. KneeKG assessments have also characterised patients dissatisfied because of pain after arthroplasty, identifying modifiable markers such as stiff‐knee gait, valgus collapse during walking, or excessive external tibial rotation [26, 29]. This is clinically relevant because the ability to perform daily activities, and especially to restore gait, ranks among the outcomes valued most by both patients and surgeons [64].

Interestingly, preoperative knee functional deficiencies can also still be present post‐TKA, as these may be due to compensatory neuromuscular habits assimilated with time [31, 43]. In these cases, the replacement of articular surfaces alone may not allow a reset of biomechanical patterns which have developed throughout the years of OA progression. This supports the need for F‐F postoperative rehabilitation, considering that preoperative altered gait mechanics persisting after surgery, especially in the sagittal plane, strongly influence joint loads and may in turn lead to prosthetic loosening [35]. Implant survivorship is also directly linked to abnormal knee kinematics, which constitute a main cause of residual pain and revision [9]. A postoperative KneeKG exam is therefore most informative once other causes of limitation have been excluded (e.g., infection, malalignment or metal allergy).

KneeKG functional data constitute actionable targets that can be addressed by non‐surgical interventions such as neuromuscular exercises, gait training, bracing, and orthotics [12, 59]. The combination of education and tailored exercises targeting the identified functional deficiencies led to better clinical outcomes (symptoms, pain, function and satisfaction), higher treatment adherence, and correction of functional deficits compared to conventional OA management [11, 13, 28]. This was supported in recent clinical practice guidelines (CPG) published by the AAOS which recommend education as a central tool, citing a recent randomised controlled trial on a personalised approach based on education coupled with KneeKG measures to improve outcomes in non‐surgical OA patients [2, 13].

This conservative strategy has since been adapted to the postoperative context. The objectives are the same, the assessment and correction of kinematic markers linked to outcomes and satisfaction rather than relying on static imaging data to guide treatment. Recent proceedings communicated promising results of a KneeKG (or kinematic) guided rehabilitation approach following TKA [56, 57]. A group of patients benefited from a F‐F rehabilitation programme including a KneeKG exam 3 months after surgery, combined with an education session and an individualised home‐based exercise programme targeting the identified functional deficiencies. They had access to an online exercise platform and a non‐mandatory follow‐up call 2 weeks later (to adjust the exercises if necessary). These patients reported significant improvements in KOOS‐JR (+9.8, *p* < 0.001) leading to higher scores at 6 months compared to controls who followed standard rehabilitation (79.6 vs. 70.1, *p* = 0.004). Almost all F‐F patients (94%) met the patient acceptable symptom state (PASS) criteria 6 months after TKA (46% for controls, *p* = 0.001) suggesting a faster recovery, and demonstrated excellent satisfaction levels (100% vs. 78%, *p* = 0.04). The sub‐group of patients who completed a follow‐up more than 1‐year post‐TKA (16.4 months on average), without any additional visit except the one at 3‐month, maintained and even amplified their KOOS‐JR improvements (+16.0 pts from baseline KneeKG exam, latest KOOS‐JR = 86.4). Patients further reported fewer mild to moderate walking limitations (*p* = 0.04), supporting the integration of functional KneeKG data to guide personalised postoperative care. In addition to driving faster recovery and better outcomes, KneeKG assessments conducted a few months apart can allow more accurate and objective monitoring of patients' functional evolution, and may also constitute decisive information for surgeons who need to document the impact of treatments.

### KneeKG versus other motion‐analysis systems

Visual gait assessment, performed live or on video, remains the most cost‐effective and widely available option in clinic. It can identify global dysfunctions, but accuracy and reliability are major limitations when clinicians need objective quantification of specific biomechanical markers across the gait cycle. Historically, precise quantification has therefore relied on gait laboratories using reflective marker‐based motion capture, which advanced understanding of the links between knee disorders and biomechanical dysfunctions; however, these assessments are time‐consuming and poorly accessible for routine care because of high operational costs, the need for trained personnel, and the burden of data processing and interpretation [15]. Markerless vision‐based systems may represent the ultimate goal to maximise accessibility while maintaining accuracy [48]. Wearable inertial sensors offer practicality and lower cost, and can monitor spatiotemporal parameters and gross range of motion, but evidence suggests limited accuracy for clinically actionable 3D alignment and kinematic variables needed for surgical planning [38]. In this context, KneeKG can be viewed as a pragmatic middle ground, aiming to combine clinic feasibility with objective, knee‐specific, weight‐bearing 3D kinematic marker assessment, consistent with its intended role in early reports [48].

### Surgeon's perspective

In daily practice, KneeKG can be used as a functional adjunct to imaging modalities to characterise dynamic alignment, thrust, flexion contracture, pivot pattern and tibiofemoral translation before surgery. These markers provide information on alignment philosophy, bearing selection and soft‐tissue strategy (particularly mid‐flexion balance), helping clinicians to frame preoperative counselling. Intraoperatively, the preoperative kinematic profile could be translated into target gaps and component aims with future navigation or robotics to anticipate coronal behaviour and reduce on‐table surprises. At the moment, these functional data are not directly embedded within a surgical navigation or robotic system, the KneeKG assessment being a complementary clinical exam. Since it can be done in clinic, it easily blends in the patient's pathway before surgery (i.e., same day as the appointment with anaesthetist, radiographs, etc.).

While results have to be integrated by surgeons themselves into their workflow in the same way as CT or X‐ray outputs, future applications should consider facilitating their integration, especially in digital surgeries, to enable more orthopaedists to include these data in their decision‐making. Postoperatively, the exam audits whether plans have delivered the intended dynamic behaviour, provides functional causes of residual symptoms (e.g., persistent thrust or stiff‐knee gait) and directs function‐focused rehabilitation programmes. Across the care pathway, shared WB metrics provide a common language for surgeons, therapists and patients, supporting expectation‐setting and enabling iterative improvement from case to case.

### KneeKG's limitations

Several limitations should be considered when interpreting KneeKG findings and translating them into clinical decisions. First, KneeKG remains an external tracking method; although the exoskeleton concept aims to reduce soft‐tissue artefacts compared with skin‐mounted markers, residual artefacts and registration errors may persist, particularly for transverse‐plane variables and anteroposterior translation, and outputs can be sensitive to landmark digitisation and technician‐dependent setup. Second, the exam typically relies on standardised tasks (most commonly treadmill walking), which improves repeatability but may not fully reflect overground gait or higher‐demand activities (stairs, uneven terrain, turning), limiting ecological validity for some patients. Third, KneeKG primarily provides kinematics and derived markers; it does not directly measure kinetics, muscle activation, or contact mechanics, and therefore cannot by itself distinguish whether an abnormal pattern is driven by pain inhibition, weakness, compensation, or structural constraint. Finally, feasibility and generalisability may be reduced in specific subgroups (very limited walking capacity, discomfort with the device, extreme body habitus).

## CONCLUSION

Since the 2012 technical review, a growing evidence base has shown that the KneeKG enables routine integration of weight‐bearing 3D kinematics into the care of patients undergoing TKA. While the 2012 literature largely established the device's technical principles, feasibility, and validity, more recent work has shifted the focus toward clinical implementation across the episode of care, using KneeKG data to inform alignment and balancing strategy including in conjunction with contemporary navigation, to define patient‐specific functional targets, to audit whether surgical plans deliver the intended dynamic behaviour, and to direct targeted, function‐focused rehabilitation. By aligning preoperative planning and intraoperative execution with objective weight‐bearing function, KneeKG offers a pragmatic route to more reliable restoration of function and improved patient‐reported outcomes after TKA.

## AUTHOR CONTRIBUTIONS

**Clément Favroul**: Study design; manuscript writing. **Alix Cagnin**: Study design; manuscript writing. **Cécile Batailler**: Manuscript editing. **Sébastien Lustig**: Study design; supervision; literature review and manuscript editing. All authors read and approved the final manuscript.

## CONFLICT OF INTEREST STATEMENT

Clément Favroul declares no conflicts of interest. Alix Cagnin is an engineer at Emovi (manufacturer of KneeKG). Cécile Batailler consultant for Stryker, Smith & Nephew. Sébastien Lustig consultant for Stryker, Smith & Nephew, Heraeus, Depuy Synthes; Institutional research support from Groupe Lepine, Amplitude; Editorial Board for *Journal of Bone and Joint Surgery* (Am).

## ETHICS STATEMENT

The authors declare that ethics statement is not required for this study.
